# Second-Generation Antiandrogens: From Discovery to Standard of Care in Castration Resistant Prostate Cancer

**DOI:** 10.3389/fonc.2019.00801

**Published:** 2019-08-28

**Authors:** Meghan A. Rice, Sanjay V. Malhotra, Tanya Stoyanova

**Affiliations:** ^1^Department of Radiology, Canary Center at Stanford for Cancer Early Detection, Stanford University, Palo Alto, CA, United States; ^2^Department of Radiation Oncology, Stanford University, Palo Alto, CA, United States

**Keywords:** prostate cancer, antiandrogens, CRPC, second-generation antiandrogens, enzalutamide, apalutamide, abiraterone acetate, darolutamide

## Abstract

Prostate cancer is the most commonly diagnosed cancer affecting men in the United States. The prostate is a hormone-dependent gland in which androgen hormones testosterone and dihydrotestosterone bind to and activate the androgen receptor, initiating nuclear translocation of androgen receptor and a subsequent signaling cascade. Due to the androgen dependency of the prostate, androgen deprivation therapies have emerged as first line treatment for aggressive prostate cancer. Such therapies are effective until the point at which prostate cancer, through a variety of mechanisms including but not limited to generation of ligand-independent androgen receptor splice variants, or intratumoral androgen production, overcome hormone deprivation. These cancers are androgen ablation resistant, clinically termed castration resistant prostate cancer (CRPC) and remain incurable. First-generation antiandrogens established androgen receptor blockade as a therapeutic strategy, but these therapies do not completely block androgen receptor activity. Efficacy and potency have been improved by the development of second-generation antiandrogen therapies, which remain the standard of care for patients with CRPC. Four second-generation anti-androgens are currently approved by the Food and Drug Administration (FDA); abiraterone acetate, enzalutamide, and recently approved apalutamide and darolutamide. This review is intended to provide a thorough overview of FDA approved second-generation antiandrogen discovery, treatment application, strategies for combination therapy to overcome resistance, and an insight for the potential future approaches for therapeutic inhibition of androgen receptor.

## Introduction

Prostate cancer (PC) has long been the most commonly diagnosed non-cutaneous cancer in men in the United States, and currently has the second highest cancer-associated deaths after lung cancer (1). Through the process of annual digital rectal exams (DREs), prostate specific antigen (PSA) screening and follow-up biopsies, PC is often diagnosed in an actionable time-frame. When diagnosed early, PC grows slowly, and therefore, men can be monitored *via* active surveillance (also termed “watchful waiting”) for some time prior to medical intervention to limit overtreatment and increase quality of life. Localized PC is primarily treated by either radical prostatectomy and/or radiation therapy and is often well-managed on these regiments. Metastatic or recurrent PC is usually treated with hormonal therapy, or androgen deprivation therapy (ADT), also termed therapeutic castration. These therapeutic interventions work by inhibiting the testosterone production of the testes and prostate tumors blocking AR, which is largely impactful as over 80% of PC is androgen dependent (2). ADT includes luteinizing hormone-releasing hormone (LHRH) agonists and antagonists, and AR blockers such as bicalutamide. Patients undergoing ADT have excellent initial responses, however most cases result in relapse within a few years due to alternative mechanisms of androgen receptor (AR) signaling, AR amplification or alternative splicing, intratumoral androgen production, or adrenal gland testosterone production at which time the disease is termed castration resistant PC (CRPC) and it is currently incurable (3).

CRPC is often metastatic and is responsible for the majority of PC-associated deaths. Treatment options for CRPC are antiandrogen therapies, taxane-based chemotherapies, sipuleucel-T (provenge) vaccine, or radium-223. Antiandrogens differ from LHRH antagonists by blocking specific aspects of androgen signaling. The first-generation antiandrogens bicalutamide, nilutamide, or flutamide exclusively target AR translocation to the nucleus and prevent downstream signaling, while second-generation antiandrogens enzalutamide, apalutamide and darolutamide improve upon this mechanism, and abiraterone acetate prevents androgen biosynthesis.

**Figure 1:**
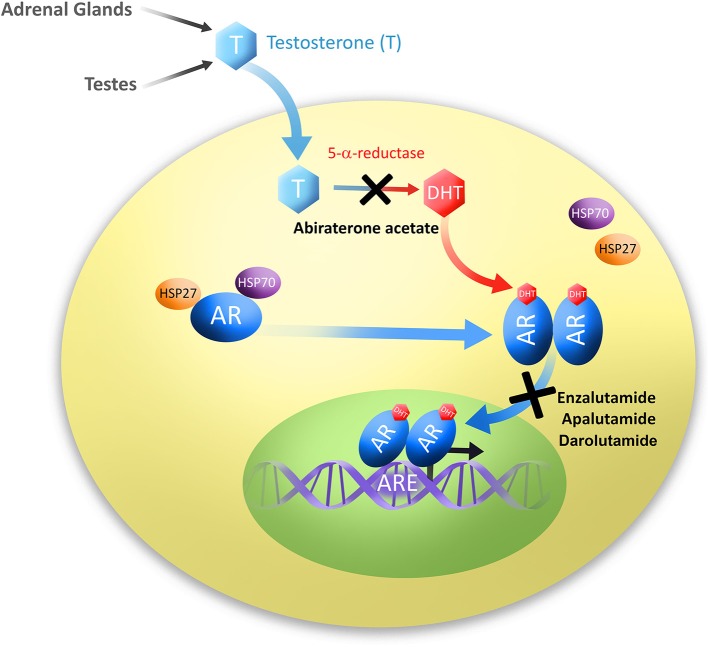
Diagram of androgen production and subsequent signaling through the androgen receptor. Testosterone (T) is produced in the testes and adrenal glands. Testosterone is then converted to its most common and active metabolite, dihydrotestosterone (DHT) by 5α-reductase. Androgens, usually DHT, bind to the androgen receptor (AR), dissociating chaperone proteins, members of the heat shock protein family HSP27 and HSP70. Ligand-bound AR molecules homodimerize and translocate to the nucleus where they bind to androgen response elements (ARE), and act as transcription factors to signal downstream targets. Second-generation antiandrogens are illustrated at their points of pathway disruption; Abiraterone acetate prevents androgen biosynthesis, and Enzalutamide, Apalutamide and Darolutamide prevent AR translocation to the nucleus.

The original discovery of a link between androgen ablation and prostatic disease was made in 1786 by John Hunter who demonstrated effects of surgical castration on animals and secondary sex organ sizes (4, 5). However, it was not until 1941 that Charles Huggins and Clarence Hodges discovered androgen deprivation to be an effective treatment of PC (6). In contrast to prostate biology, studying the AR has only reached thorough understanding in the past 30 years, with the first sequence of AR being cloned and mapped to the X chromosome in 1988 by Lubahn et al. (7) Canonical signaling through the AR occurs by androgen ligand activation of the AR (Figure 1). The most common of which are testosterone and its derivative dihydrotestosterone (DHT) which is converted by 5-alpha-reductase, though DHT binds AR with 2–5 times higher affinity than testosterone (8, 9). AR is composed of four distinct domains: the N-terminal domain, DNA binding domain (DBD), a hinge region which allows for N- and C-terminal interaction, and a C-terminal ligand binding domain (LBD) (10). Prior to activation, AR is located in the cytoplasm bound to several chaperone proteins, members of the heat-shock protein family (Figure 1). Androgens bind to the AR ligand binding domain releasing AR chaperones and allowing AR to homodimerize and translocate to the nucleus where it acts as a transcription factor for androgen responsive genes such as PSA and others (Figure 1). Many aspects of AR signaling allow for therapeutic exploitation, such as sequestration of DHT ligands that activate AR, blockade of AR N-C terminal interaction, disruption of AR co-activator interaction, and prevention of AR nuclear translocation (11, 12).

The first therapeutic attempts at AR inhibition were the development of first-generation antiandrogens; that is, steroidal analogs meant to block AR ligand activation. However, developed resistance to these inhibitors became quickly apparent. It is believed that the primary drug resistance mechanisms arise from AR amplification, point mutations, expression of AR splice variants which are ligand independent, intratumoral androgen production or downstream signaling mechanisms (3). It was also determined that in the presence of excess AR, as is commonplace in the context of aggressive PC, first-generation AR antagonists undergo a switch acting as agonists promoting tumor progression in preclinical models of PC (13). This agonist switching results in increased AR mRNA and protein levels, leading to resistance to antiandrogen therapy (14). These changes in pharmacological properties led to the need for second-generation antiandrogens, specifically looking to compounds that would retain antagonistic properties when exposed to excess AR. Further, first-generation antiandrogens are almost completely penetrable by AR mutations, and in the event of bicalutamide discontinuation, a common observation is androgen withdrawal syndrome in which tumors would regress due to AR mutations and agonistim of bicalutamide (15).

Prior to development of second-generation antiandrogens, the most effective treatment strategy for CRPC included taxane chemotherapeutic agent docetaxel, and subsequently cabazitaxel (16, 17). Another therapy option included immunotherapy with the sipuleucel-T (Provenge) vaccine. Sipuleucel-T is the first dendritic cell-based cancer vaccine based on Dr. Edgar G. Engleman's approach in which patient dendritic cells are harvested and pulsed with recombinant prostatic acid phosphatase fusion protein (PAP) and administered back to the host, stimulating autoimmunity to the PC (18). Sipuleucel-T was ultimately approved by the FDA on April 29, 2010 after the success of clinical trials (19).

The development and FDA approval of second-generation, or next-generation antiandrogen treatments has since significantly altered the armamentarium of PC, specifically in treatment of CRPC. Second-generation antiandrogens have increased specificity to the androgen receptor over other steroidal receptors, act at a higher affinity than the previous generation, are exclusively antagonistic to the AR, and in turn elicit no androgen withdrawal syndrome. These compounds have greatly increased patient lifespan, extended metastasis free overall survival, decreased circulating and intratumoral androgens and serum PSA. Even with these advances CRPC has not been eradicated. It is important to fully understand the successes and downfalls in implementation of antiandrogen therapy to patients to look to the future of CRPC treatment.

## Androgen Biosynthesis Inhibition

In contrast to androgen receptor blockade, another method of androgen signaling inhibition is the upstream blockade of androgen production. Androgens are produced in the testes, the adrenal glands as well as intratumorally. They are processed by the cytochrome p450 enzyme 17R-hydroxylase-17,20-lyase (CYP17A1). Androgens are then released and circulate through the body. Targeting the biosynthesis mechanism of testosterone through inhibition of CYP17A1, also produced in the testes and adrenal glands, would inhibit production of DHT and decrease endogenous androgen levels. The FDA approved abiraterone acetate will be described in this section, but it is not the only member of this class of inhibitors. Ketoconazole, an FDA approved antifungal compound is also an androgen biosynthesis inhibitor. However, ketoconazole has toxicity due to non-specificity to CYP17A1, though it has also been tested as treatment in PC (20).

Other compounds that have been tested as biosynthesis inhibitors for PC include orteronel (TAK-700), a non-steroidal inhibitor of 17,20-lyase that was tested in clinical trials for metastatic CRPC, as well as galeteronel/TOK-001/VN-124. Galeteronel is a unique dual androgen antagonist and biosynthesis inhibitor that was well-tolerated in Phase 1 trials (21, 22). However, neither compound was able to meet their clinical trial endpoints and have been since terminated, leaving abiraterone acetate as the only currently approved compound in this class for CRPC treatments (23, 24).

### Abiraterone Acetate

**Other names**: Zytiga, Yonsa.

#### Discovery

The discovery of abiraterone, predecessor to abiraterone acetate, was fueled by the potential of CYP17 inhibition as a treatment for PC. Previously the antifungal ketoconazole which also inhibits CYP17 was determined to inhibit PC growth, with the limitations of low potency, causing adrenal insufficiency, and a general short half-life in the body (25, 26). Abiraterone/CB7598 was a success in that it decreased testosterone production throughout the body and did in fact target CYP17. Abiraterone underwent clinical trials for treatment of advanced PC. However, abiraterone had high toxicity due to the non-specific inhibition of other members of the CYP family. In an attempt to limit toxicity, 20 new compounds were synthesized with variations on the original abiraterone structure to ultimately inhibit the side effects of treatment associated with steroidal hormone interactions. The steroidal scaffold of abiraterone was replaced with non-steroidal cores. One of the compounds, abiraterone acetate, was among them, demonstrating comparable activity to abiraterone with even higher selectivity for CYP17 (27–29).

Abiraterone acetate was the first of the second-generation antiandrogens to be approved by the FDA in April 2011 (Table 1). In contrast to canonical antiandrogens which target androgen receptor, abiraterone acetate is an androgen biosynthesis inhibitor, and the only one approved by the FDA for use in PC at this time (Figure 1 and Table 1) As such, it targets cytochrome P450 enzyme 17R-hydroxylase-17,20-lyase (CYP17). CYP17 processes testosterone and is produced in the testes and adrenal glands. Therefore, inhibition of CYP17 prevents androgen production in both locations and was therefore predicted to be more effective in androgen dependent PC than Gonadotropin-releasing hormone (GnRH) analogs (ADT).

**Table 1:** Pharmacological and clinical properties of FDA approved second-generation anti-androgens.

	**Compound**	**Target**	**IC50**	**Adverse effects**	**Applications**	**FDA pipeline**
Biosynthesis Inhibitors	Abiraterone acetate	CYP17	72 nM (cell free) (27)	Hypertension, hypokalemia, edema, hepatotoxicity, adrenocortical insufficiency (33)	AAP for metastatic CRPC, and metastatic high-risk castration-sensitive PC (33–35, 37–39)	Approved
Androgen Receptor Blockers	Enzalutamide	AR antagonist	36 nM (LNCaP cells) (40)	Fatigue, hypertension, hot flush, dizziness, nausea and falls; elevated risk of seizure (41)	non-metastatic CRPC, metastatic CRPC (41–47)	Approved
	Apalutamide	Selective and competitive AR inhibitor	16 nM (cell free) (48)	Fatigue, hypertension, rash, diarrhea, nausea, weight loss, arthralgia, fall, hot flush, decrease in appetite, features and peripheral edema (49, 50)	non-metastatic CRPC (49–53)	Approved
	Darolutamide	AR antagonist	26 nM (AR-HEK293 cells) (54)	Fatigue, nausea, pain in extremities, rashes, ischemia and heart failure (55–58)	non-metastatic CRPC (58)	Approved

#### Clinical Trials and Path to FDA Approval

Phase 1 and 2 studies were performed on a small cohort of 30 chemotherapy-naïve patients, some of which had previously undergone ketoconazole treatment (NCT00473746) (30–32). No dose-limiting toxicities were observed, and many patients benefited from decreased circulating androgens (33). This study found that the main side effects of abiraterone acetate were associated with low levels of mineralocorticoids, including hypokalemia, fluid retention, and hypertension. These events are ameliorated in combination with glucocorticoids such as prednisone, which has become a standard combination therapy.

For metastatic PC, abiraterone acetate was tested in chemotherapy refractive PC (COU-AA-301; NCT00638690) and chemotherapy-naïve patients (COU-AA-302; NCT00887198). COU-AA-301 enrolled 1,195 patients over 147 sites in 13 countries. Patients with metastatic CRPC were included with progression following chemotherapy on docetaxel, and observed an overall increase in median overall survival from 10.4 months in placebo controlled group to 15.4 months (34, 35). Progression free survival increased as measured by PSA (increased from 6.6 to 8.5 months), and radiologic progression (increased from 3.6 to 5.6 months) (35). In COU-AA-302, abiraterone acetate and prednisone were provided for metastatic CRPC patients who had not previously failed chemotherapy and was also highly effective (36–38). In this trial of 1,088 enrolled patients, overall survival increased from 10.9 to 14.8 months, time to PSA progression increased 6.6 to 10.2 months, and progression-free survival increased 3.6 to 5.6 months compared against placebo-prednisone group (36).

While abiraterone remains an effective clinical therapy to date, new discoveries are still underway to further optimize effectiveness, harnessing more active metabolites found in CRPC patients as primary therapies such as in the case of Δ^4^-abiraterone (D4A) (39).

## Androgen Receptor Blockers

The most utilized mechanism of antiandrogens is blockade of AR signaling by sequestration of AR itself, thus preventing nuclear translocation and subsequent signaling to AR target genes (Figure 1 and Table 1). While first-generation antiandrogens also fit this classification, as a group, second-generation androgen receptor blockers no longer exhibit agonist/antagonist switch, androgen withdrawal syndrome, and have decreased patient toxicities. The main new side effect associated with androgen receptor blockade is increased risk of seizure. This is due to penetrance of the compounds through the blood-brain barrier (BBB) and subsequent inhibition of the γ-aminobutyric acid receptor (GABA_A_). Enzalutamide discussed herein was the first of the class to be characterized and has the highest risk of seizures at low doses. The recently approved apalutamide and darolutamide provide reductions in brain penetrance and reduced association with clinical risk of seizure, demonstrating promising advances in the field of antiandrogen therapy.

### Enzalutamide

**Other names**: MDV3100, XTANDI.

#### Discovery

Enzalutamide was the first characterized second-generation antiandrogen. Developed in the laboratories of Drs. Charles Sawyers and Michael Jung, enzalutamide was isolated from a mutagenic screen of non-steroidal agonist RU59063 (40). Compounds were tested for their potential as antagonists of AR and then optimized based on stability and bioavailability. These studies led to the discoveries of MDV3100 and RD162, which demonstrated 5-8-fold greater affinity to AR, and only slightly less affinity than AR ligand DHT. Enzalutamide also inhibits nuclear translocation of AR, as well as inhibits AR DNA binding and coactivator recruitment (Figure 1 and Table 1) (40). Importantly, these compounds did not exhibit agonistic behavior to AR when used in an AR saturated environment in contrast to their first-generation predecessors (40). Ultimately enzalutamide was prioritized over RD162 for clinical development based on favorable drug properties (59).

#### Clinical Trials and Path to FDA Approval

In 2012, enzalutamide was approved by the FDA for the treatment of men with metastatic CRPC (Table 1). Enzalutamide exhibited greater overall survival over placebo treated patients in clinical trials of patients who relapsed on chemotherapy (AFFIRM trial; NCT00974311), from 13.6 to 18.4 months, as well as radiographic progression-free survival in first line therapy for chemotherapy-naïve patients (PREVAIL trial; NCT01212991), increased from 14 to 65% (42, 43). In the PREVAIL study, enzalutamide was beneficial to patients irrespective of visceral disease, or metastatic sites (low or high volume bone disease as well as lymph node only metastasis) (44). In a head-to-head trial (STRIVE trial; NCT01664923), enzalutamide and first-generation antiandrogen bicalutamide were directly compared. In this trial, treatment with enzalutamide significantly reduced the risk of PC progression and PC-associated death compared to bicalutamide in both metastatic and non-metastatic CRPC (45). FDA approval of enzalutamide was later expanded to include non-metastatic CRPC following positive results of the Phase 3 PROSPER trial (NCT020032924), demonstrating improved metastasis-free survival, which has largely replaced overall survival as a more accurate representation of disease progression in aggressive PC (46, 47).

An advancement from first-generation antiandrogens to enzalutamide was the observation that there was no observed androgen withdrawal syndrome following treatment with enzalutamide (60). Clinically enzalutamide was generally well-tolerated, and the most common side effects included fatigue, arthralgia, and constipation (41). One new complication associated with high dosages of enzalutamide is a risk of increased seizures stemming from the binding to and inhibition of the γ-aminobutyric acid receptor (GABA_A_), which was an important object of investigation in development of later therapies such as apalutamide (41).

### Apalutamide

**Other names**: ARN-509, JNJ-56021927, Erleada.

#### Discovery

Building upon the knowledge gained from development of enzalutamide, a more recent addition to the antiandrogen class was apalutamide, which is also a next-generation AR antagonist. Apalutamide was demonstrated to bind with high affinity to the ligand-biding domain of the AR leading to inhibition of its translocation to the nucleus and DNA binding. Apalutamide is a synthetic compound generated based on structure-activity relationship (SAR)-guided chemistry (Figure 1 and Table 1). This was important in identifying compounds which would remain completely antagonistic in the presence of AR expression. Apalutamide is a biaryl thiohydantoin and its selection as candidate was based upon assessment of antagonistic vs. agonistic activity of AR in LNCaP cells overexpressing AR (48). *In vitro*, apalutamide bound AR with 7–10 fold higher affinity than bicalutamide with an IC50 value of 150 nmol/L. Apalutamide binds AR in the same ligand-binding domain as bicalutamide but with greater affinity, and unlike bicalutamide, retains full antagonist activity in the setting of AR overexpression (48). Furthermore, apalutamide was shown to be selective for AR over other nuclear hormone receptors (48). Ultimately apalutamide was considered an ideal candidate for preclinical assessment having a long serum half-life, low systemic clearance and high oral bioavailability, while demonstrating dramatic effects in reduction of tumor volume in preclinical models of CRPC (48). Further, apalutamide was also effective on non-castrated animals in preclinical studies suggesting it may also be effective prior to castration resistance.

The advantages of apalutamide over enzalutamide included greater efficacy and higher tumor to plasma ratio and four-fold lower concentrations in the central nervous system, suggesting its decreased ability to permeate the blood brain barrier (BBB), potentially indicating a lower risk of seizure activity (48).

#### Clinical Trials and Path to FDA Approval

Phase I testing was performed on a small cohort of 30 patients with metastatic CRPC and was well-tolerated at all doses (51). Ultimately, the dose at 240 mg/d was selected for further testing in phase II studies. This study was successful based on responses in PSA levels and disease control of patients, delaying time to cancer progression. After selection of patient dose from the Phase I trial, it was proceeded by a multicenter phase 2 study enrolling non-metastatic CRPC at risk of progression as defined by a PSA ≥ equal to 8 ng/ml or PSA doubling time <10 months (NCT01171898) (52). The study was deemed highly successful noting responses in PSA in addition to prolonged metastasis free survival and used to support the design of the phase 3 apalutamide SPARTAN trial (NCT01946204) (52, 53). The SPARTAN trial was an enormous international undertaking spanning 332 centers across 26 countries with 1,207 patients (53). Apalutamide increased metastasis free survival from 16.2 to 40.5 months, with results favorable enough to unblind the trial and offer placebo patients the new therapy. Based on these data apalutamide was approved under priority FDA review for non-metastatic CRPC in 2018 (Table 1).

The safety of apalutamide for metastatic CRPC was evaluated in patients who had either undergone treatment with abiraterone acetate plus prednisone (AAP), or AAP-naïve patients (ARN-509-001; NCT01171898). Apalutamide was again safe, well-tolerated, and demonstrated therapeutic benefit in in patients with metastatic CRPC. Apalutamide treatment was incredibly effective in patients naïve for AAP treatment with an 80% reduction in PSA decline compared to a 22% decline in post-AAP therapy patients (49). The TITAN trial was designed to test apalutamide in metastatic CRPC, newly diagnosed without prior therapies (NCT02489318). Similar to the non-metastatic trial, the TITAN study has recently been unblinded to allow patients to switch to apalutamide treatment based on improvements to progression-free survival as well as overall survival (50).

### Darolutamide

**Other names**: ODM-201, BAY1841788, NUBEQA.

#### Discovery

Darolutamide is a unique AR antagonist generated as part of a synthetic molecule library, specifically interested in inhibiting AR nuclear translocation (Figure 1 and Table 1). In competitive AR binding assays darolutamide or the active metabolite (ORM-15341) markedly increased potency over enzalutamide and apalutamide in Ki and IC50 values (54). Uniquely, darolutamide and ORM-15341 each individually inhibited wild-type AR as well as clinically relevant AR mutations AR(F876L), which trigger enzalutamide and apalutamide antagonist to agonist switch, as well as AR(W742L) and AR(T877A) which cause bicalutamide agonist switch (54). This inhibition of all known AR mutations is a large advancement for the field of PC treatment that may lead to decreased instance of therapeutic resistance (61). Further, darolutamide inhibited *in vivo* growth of VCaP CRPC xenografts, and exhibited low penetrance of the BBB in mice and rats (54, 62).

#### Clinical Trials and Path to FDA Approval

Phase 1 and 2 studies tested safety in patients with CRPC, performed in the ARADES (55, 56) (NCT01429064) and ARAFOR (NCT01784757) (57) trials [and reviewed in (63)]. Darolutamide proved safe and well-tolerated, in addition to reducing tumor volume and PSA levels in both trials (55–57). Darolutamide was approved by the FDA on July 30, 2019 with fast track designation for use in non-metastatic CRPC, making it the most recently FDA approved PC therapy. Approval was granted based on performance in the ARAMIS trial (NCT02200614) (58). The study encompassed 1,509 patients as a multicenter, double-blind tral. Metastasis free survival was 40.4 months in darolutamide treated patients compared to 18.5 months in placebo treated patients. Current clinical recommendation is that darolutamide treatment be combined with ADT or bilateral orchiectomy for non-metastatic CRPC. Darolutamide provides promising reductions in brain penetrance, as well as effectively inhibiting all known AR mutations (61). Ongoing trials include the phase 3 ARASENS trial in which darolutamide plus ADT are combined for treatment of metastatic hormone-sensitive PC (NCT02799602), with an anticipated endpoint in 2022.

## Resistance and Combination Therapy Strategies

### Resistance

Therapeutic resistance is not a new problem specific to antiandrogen therapy. Resistance mechanisms that evade therapy are a consistent medical challenge in ailments from oncology, in solid cancers and hematological malignancies, to viral infections such as human immunodeficiency virus (HIV). The first instance of clinical combination therapy was in HIV as an answer to the rapid rate of mutation of the disease-causing retrovirus. Termed highly active antiretroviral therapy (HAART), it combined multiple antiretroviral drugs preventing the growth of the virus, and is still used to date (64). Early targeted cancer therapies had great success such as in treatment with Gleevec (imatinib) in leukemia, which was thought to be a “magic-bullet” for cancer therapy (65). While still widely utilized, Imatinib commonly results in resistant disease (66). Oncogenic treatments have begun following a similar trend to HAART therapy to prevent cancer resistance or sensitize to already refractory therapies by targeting multiple pathways essential to oncogenesis such as growth and angiogenesis (67).

Since the treatment with first-generation antiandrogens, resistance associated with androgen withdrawal has been commonly characterized. Greater than 95% of testosterone production in the male body is generated in the testes, yet adrenal, prostatic, and intratumoral androgens play a great role in resistance as minute overexpression of AR is enough to counteract androgen withdrawal, sensitizing tumors to small amounts of androgen to activate AR signaling [reviewed (68–70)]. Further, genetically one-third of CRPC patients have AR amplification (71).

Mutation is one common mechanism by which androgen withdrawal experiences resistance. It was first reported in 2003 by Hara et al. who discovered a mutation of AR which could cause resistance to bicalutamide by allowing an AR antagonist to agonist switch (15). Several AR point mutations associated with first-generation antiandrogens include T877A association with flutamide resistance (15), as well as W741C associated with bicalutamide agonistic switch (72). Subsequently, other clinically relevant mutations of AR (F876L and F877L) were also found to cause an AR agonistic response in enzalutamide and apalutamide, respectively (73, 74). Darolutamide has clinically shown to act as a full antagonist toward AR F876L among other mutations, making a strong platform for darolutamide to quickly gain traction as standard of care in non-metastatic CRPC now that it received FDA approval (54). One method of abiraterone resistance comes from intratumoral generation of androgens and CYP17A1 production, selecting for increased intratumoral production, in conjunction with a progesterone responsive mutant AR (T877A). These tumors are still responsive to steroids and may benefit from switching therapies to androgen receptor blockade (75). Abiraterone treatment increases progesterone, activating AR mutations previously associated with flutamide resistance (T878A) (76).

While second-generation antiandrogens have taken a huge stride in treatment of advanced PC, 20–40% of patients still do not respond to these therapies, as measured by PSA (34, 36, 42, 77), and of the patients that respond, resistance will undoubtedly occur given enough time. Androgen receptor splice variants, such as AR-V7 commonly occur in clinical specimens, and approximately 75% of CRPC cases (78, 79). AR splice variants missing the ligand binding domain signal independently of AR ligand stimulation and are therefore among the most common resistance mechanisms of antiandrogen therapy (80). Ligand binding mutations have been characterized in as many as 20% of patients who have progressed following first-generation antiandrogen treatment (81, 82). Current diagnostic testing can determine AR-V7 expression among other biomarkers from patient needle biopsies (oncotype Dx) to predict a Genomic Prostate Score (GPS) associated with aggressive PC for clinical decision-making (83–85). Further, other studies have followed AR-V7 levels in circulating tumor cells of metastatic CRPC patients and correlated with risk of recurrence, with follow-up PSA serum levels (80).

Mechanisms which have been proposed to prevent therapeutic resistance include drug cycling, intermittent hormonal therapy as opposed to continuous, and combination therapy.

### Combination Therapy Strategies

Combination therapies have become more common in recent years in many medical fields, especially oncology. Due to resistance mechanisms, including those just described, antiandrogens are often not prescribed as a single therapy. Antiandrogens were originally given in combination with ADT in a combined androgen blockade (CAB). Prior to the development of second-generation antiandrogens, first-generation antiandrogens were already being combined with medical androgen suppression. A large meta-analysis performed on data from PC patients who received combined androgen receptor blockade consisting of androgen suppression with a first-generation antiandrogen noted a mild improvement in 5-year survival (86). Another strategy in CAB entails combination of androgen receptor blockers with biosynthesis inhibitors. The reasoning behind which being abiraterone acetate working through a different mechanism may sensitize enzalutamide resistant cancers. Enzalutamide plus abiraterone was tested in patients undergoing resistance to enzalutamide in the PLATO trial (NCT01995513) under the assumption that inhibiting androgen synthesis would sensitize patients to enzalutamide (87). Abiraterone acetate with prednisone has also been safely tested with apalutamide successfully in clinical trial with observed antitumor effects in ongoing clinical trials of metastatic CRPC (NCT02257736) (88, 89). Apalutamide with standard ADT is also ongoing in the TITAN trial for metastatic CRPC and to date has improved progression-free survival as well as overall survival vs. ADT alone (90). Finally, in LATITUDE trial, Abiraterone acetate plus prednisone combined with ADT lengthened time of progression free survival 14.8–33 months (NCT01715285) (91).

In contrast to other more rapidly dividing cancers, chemotherapy for PC treatments is usually performed later in the disease progression. However, in late stage PC, taxane chemotherapy is a common standard of care. Chemotherapy has long since been given in conjunction with ADT such as in the CHAARTED trial specifically treating docetaxel with ADT for patients with high volume disease (NCT00309985) (92). Looking forward, chemotherapy treatment is now being tested with antiandrogens. Such as in the STAMPEDE trial; a unique multi-arm multi-stage clinical trial attempting to assay multiple treatment strategies. Within, STAMPEDE includes groups of patients receiving combination of ADT with radiotherapy, docetaxel and abiraterone and ADT with enzalutamide, abiraterone and prednisolone (NCT00268476). Another example is the treatment of apalutamide and everolimus in patients with metastatic CRPC (49).

Radiotherapy is a common treatment for localized PC as well as palliative treatment for bone metastases. Enzalutamide plus radiation *in vitro* sensitizes cells to radiation (93). In trial COU-AA-31(NCT00638690), abiraterone acetate with radiation were safely co-administered to patients with a perceived advantage in palliative bone metastasis response. Abiraterone acetate with radiotherapy has been further tested in two separate phase 3 clinical trials including ERA-223 (NCT02043678), and in the ongoing PEACE 1 trial as well, though this trial uniquely combines ADT, abiraterone, docetaxel and radiation therapy (GETUG-AFU-21; NCT01957436) (94).

Aside from the discovery of sipuleucel-T, immunotherapy for PC treatment is not a heavily researched avenue. This is largely because PC often does not have high levels of acquired genetic mutations. Even still, several cancer vaccines and immunomedics have been utilized in combination with antiandrogen treatment to date. Specifically, in second-generation antiandrogens, sipuleucel-T has undergone clinical trial in combination with abiraterone acetate plus prednisone (AAP) (P11-3; NCT01487863). In this trial sipuleucel-T was administered safely either concurrently or sequentially with AAP, and patients had similar immunologic prime-boost effects, commonly associated with effectiveness of the vaccine (95). While impact on overall survival is still to be determined, the ability to overlap two successful non-interfering therapies often has additive effects. Enzalutamide is being tested in preclinical combination with a poxviral-based metastasis vaccine which largely targets the transcription factor Twist, commonly associated with mesenchymal phenotyping and increased epithelial to mesenchymal transition (EMT) in cancer (96, 97). Finally, pembrolizumab (Keytruda), an FDA approved anti-PD-1 antibody is a well-known T-cell checkpoint inhibitor antibody commonly used in treatment of melanoma among other cancers. Pembrolizumab has undergone independent testing in two small clinical trials for metastatic CRPC with initial outcome of disease stabilization at 50% when cycles were completed (KEYNOTE-199; NCT02787005) (98). However when administered with enzalutamide, pembrolizumab demonstrated a beneficial response in PSA in 3/10 patients (NCT02312557) (99) [*Many additional clinical trials involving first-generation antiandrogens or LHRH antagonists are reviewed in* (100)].

Second-generation antiandrogens have been explored as cotreatments with therapeutics targeting other pathways important in PC growth, etc., some with very promising results. Several tested combinations include treatment with PARP inhibition exploiting mutations in DNA repair pathways. Olaparib plus abiraterone acetate increased clinical benefit in metastatic CRPC (NCT01972217) (101, 102). Abiraterone in conjunction with anti-apoptotic XIAP inhibitors exhibited success in preclinical studies (103). Cabozantinib, a kinase inhibitor with activity against MET and VEGFR2 among others, has been tested as a single agent therapy in refractory metastatic CRPC in the COMET-1 trial (NCT01605227) (104). Cabozantinib did not demonstrate a decrease in PSA levels but may be beneficial in treating and preventing skeletal burden. However, cabozantinib treatment with abiraterone acetate in metastatic CRPC exhibited strong preclinical synergy (105, 106). Abiraterone acetate with added inhibition of autophagy has synergized *in vitro* (107). Niclosamide (anti-helminthic) has strong preclinical efficacy as a PC treatment via potent inhibition of AR-V7, and sensitizes PC to abiraterone acetate, but combined with enzalutamide as a phase 1 trial had observed toxicity (108–110). Chromosomal relocations of the ETS-related gene (ERG) resulting in excess ERG signaling predominantly through joining with transmembrane serine protease 2 (TMPRSS2) (111). TMPRSS2-ERG fusions are very common in PC (111). An inhibitor of ERG, ERGi-USU inhibits ERG-positive PC cell growth, and combined with enzalutamide exhibits additive effects (112). Monoamine oxidase A inhibitors (MAOAIs), typically used to treat depression have shown to moderate PC growth and inhibit metastasis due to high concentration of MAOA in the prostate (113, 114). MAOAI combined with enzalutamide synergizes and sensitizes cells to enzalutamide treatment (115). MAOAI is also in clinical trial for non-metastatic PC clinical trials (NCT02217709). Finally, inhibitors of gamma secretase which regulates cleavage of cell surface receptors including Notch receptors among others, in combination with enzalutamide or abiraterone *in vitro* inhibits PC cell growth, migration and invasion, as well as sensitizes enzalutamide resistant cells and xenografts to enzalutamide treatment (116–119).

## Conclusions

The future of healthcare is moving toward personalized precision medicine rather than blanketed therapy to determine best practice treatment strategies for individual patients. Genomic sequencing of PC patient samples led to the discovery of mutations involving genes such as SPOP, BRCA1, or BRCA2, FOXA1, chromosomal translocations of TMPRSS2:ERG/ETS, and point mutations of AR itself (111, 120, 121). Current analyses depict the majority of PC tumors as having actionable mutations, either as aberrations, point mutations, chromosomal translocations or single nucleotide polymorphisms (SNPs) (122). Aside from AR, actionable targets represent the pathways of DNA repair, Wnt signaling, PI3K pathway, RAF kinases and CDK inhibitors.

Another shift in treatment paradigm for aggressive PC is the increased incidence of neuroendocrine PC (NEPC). It is believed the increasing frequency of NEPC in patients (currently up to 25% of CRPC patients) (123), commonly characterized by a loss of AR and increased neuroendocrine markers such as synaptophysin and chromogranin A, results from excess modulation of androgen signaling. Being AR negative, this aggressive disease is resistant to antiandrogen therapy and currently incurable. Further, a new double negative phenotype (DNPC) has been described which is negative for AR as well as the neuroendocrine phenotype (124). Rather, DNPC requires signaling through fibroblast growth factor receptor (FGFR) and the mitogen-activated protein kinase (MAPK) pathway (124). These cancers currently have no effective therapies.

New treatments targeting different aspects of the androgen receptor signaling cascade have more exploratory potential. The ideal antiandrogen compound would be a directed inhibitor toward the N-terminal AR domain inhibiting all AR isoforms described to date including full length AR as well as ligand-independent splice variants. 5α-reductase inhibitors finasteride and dutasteride are in development, and part of standard of care treatment for benign prostatic hyperplasia (BPH). However, in PC, the use of these compounds which prevent the conversion of testosterone to DHT, have been minimally implemented and could be explored further [reviewed in (125)]. Similarly, new therapies such as orteronel and galeterone are also under development, and darolutamide performed well-enough in end point clinical trials to recently receive expedited approval by FDA for non-metastatic CRPC, but may extend its treatment to more aggressive patient populations in the future. While treatment regiments for some of these compounds have not reached initial guidelines from clinical trials, these and other compounds will still continue to make their way through the clinical pipeline and hopefully alter the course of PC treatment.

Potential for antiandrogens as standalone therapeutic agents seems to have plateaued for use in advanced PC. Until such time as antiandrogens are able to inhibit all splice variants of the androgen receptor, it is far more likely that the next wave of therapeutic investigation will be focused on the combination of antiandrogen therapy with other treatments such as chemotherapy.

## Author Contributions

MR and TS conceptualized the manuscript. MR researched the literature. MR, SM, and TS wrote and reviewed the manuscript.

### Conflict of Interest Statement

The authors declare that the research was conducted in the absence of any commercial or financial relationships that could be construed as a potential conflict of interest.
